# Impact of pH on the Physicochemical, Structural, and Techno‐Functional Properties of Sesame Protein Isolate

**DOI:** 10.1002/fsn3.4760

**Published:** 2025-01-22

**Authors:** Azade Ghorbani, Ali Rafe, Mohammad Ali Hesarinejad, Jose M. Lorenzo

**Affiliations:** ^1^ Department of Food Physics Research Institute of Food Science and Technology (RIFST) Mashhad Iran; ^2^ Department of Food Sensory and Cognitive Science Research Institute of Food Science and Technology (RIFST) Mashhad Iran; ^3^ Centro Tecnológico de la Carne de Galicia Parque Tecnológico de Galicia Ourense Spain; ^4^ Área de Tecnología de los Alimentos, Facultad de Ciencias de Ourense Universidad de Vigo Ourense Spain

**Keywords:** food applications, functional, hydrophobicity, sesame protein isolate

## Abstract

Sesame protein isolate (SPI) is a highly nutritious plant protein distinguished by its essential amino acid profile. This study investigates the influence of pH on SPI's physicochemical, structural, and techno‐functional properties, highlighting its potential as a sustainable protein source for various food applications. Our findings revealed that SPI had a protein content of 90.60% and a protein extraction yield of 77.2%. The density is measured at 0.72 g/mL, with a critical compressibility index of 19.22, indicating excellent flowability for weaning foods. Notably, the ζ‐potential shifts from +39 mV at pH 3.0 to 0 at the isoelectric point (pI, 5–5.5) and becomes negative at higher pH levels. We observed a direct correlation between solubility, fluorescence intensity, and functional characteristics of SPI, with peak solubility and functional properties at acidic and alkaline pH levels and lowest values at the pI. Structural analyses confirmed the relationship between electrical charge, hydrophobicity, and functional attributes, with the highest surface hydrophobicity observed at pH 2.0. In conclusion, our findings underscore the critical role of pH in modulating the physicochemical properties of sesame protein isolate, enhancing its applicability in food formulations. SPI demonstrates significant potential as a versatile ingredient in the functional food product development.

## Graphical abstract

Sesame protein isolate (SPI) exhibited high protein extraction yield of 77.2%, making it feasible as a natural plant protein. SPI displayed unique techno‐functional properties, such as high oil holding capacity and enhanced foaming stability compared to dairy proteins, particularly at neutral pH. Findings emphasized the adaptability of SPI across various pH levels, highlighting its potential for improved applications in food formulation.

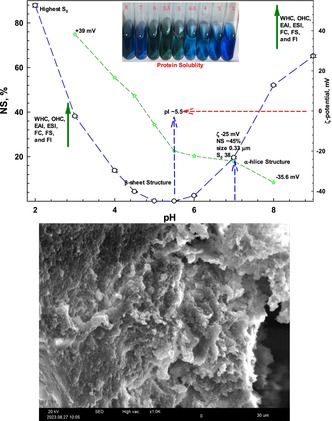

## 1 Introduction

Plant proteins have earned significant attention in recent decades due to their vital role in food security and as sustainable sources of protein (Amagliani et al. 2016). They are inexpensive, derived from unlimited supply chains, sustainable, and offer both nutritional and functional properties, which have created high demands in various food industry sectors such as bakeries, meat processing, confectionery, and beverages (Curtain and Grafenauer 2019). Ongoing research into unconventional legumes is driven by the need to address inadequate protein supplies for use as functional and nutritional supplements. Furthermore, the market for plant proteins has grown from $4.79 billion in 2019 and is expected to approach $7 billion by 2027 (Rafe et al. 2023). In contrast, agro‐industrial waste and by‐products, such as cereals, seeds, and legumes, can serve as sustainable and convenient ingredients in plant protein production (Sá, Moreno, and Carciofi 2020).

Sesame seed (*Sesamum indicum* L.) is one of the major resources of edible oil, containing 48%–55% oil and 20%–25% protein, depending on its cultivar (Morris, Wang, and Tonnis 2021; Kanu et al. 2007). It is primarily cultivated in the subtropical and tropical regions of Asia and Africa due to its high tolerance to dry conditions with global production reaching approximately 6.7 million MT in 2022 (FAOSTAT 2022). Sesame cake, a by‐product of sesame oil extraction, has a protein content that can approach 60% depending on the extraction method. This sesame protein can be utilized in food products due to its techno‐functional properties (Escamilla‐Silva et al. 2003).

Sesame protein has high nutritional value due to its essential amino acids, such as methionine and tryptophan, which distinguish it from other oilseeds and classify it as a valuable plant protein (Sá, Moreno, and Carciofi 2020; Di et al. 2022; Görgüç, Gençdağ, and Yılmaz 2020). Thus, sesame proteins can be used in combination with legumes and cereals. Mainly composed of globulins (67.3%), albumins (8.6%), prolamines (1.4%), and glutenins (6.9%), sesame proteins provide a sustainable protein source with high biological value and functional properties (Özdemir et al. 2022). The main sesame protein, identified as 11S, constitutes 60%–70% of the total proteins (Gómez‐Arellano et al. 2017) and contains various endo‐ and exo‐peptidases with high activity, predominantly found in the water‐soluble protein fraction. This makes sesame proteins a promising and versatile ingredient (Chen et al. 2021).

In order to utilize sesame protein in food formulations, it is necessary to gain more insight into the composition, physicochemical, functional, thermal, and structural properties of sesame proteins. Several studies have examined the functional properties of sesame proteins such as their emulsifying and foaming capacities (Cano‐Medina et al. 2011; Özdemir et al. 2022), their physicochemical profiles (Yüzer and Gençcelep 2023), and their protein fractions (Idowu et al. 2021). Furthermore, the polymeric structure of sesame protein can also affect consumer acceptance through its rheological properties, which have been investigated in our previous work (Rafe et al. 2023). Since the interrelation among protein composition, physicochemical, and structural properties influences its techno‐functional properties, it is worthwhile to investigate the structure–property relationships of sesame protein at different pH values. Therefore, we evaluated surface properties, including zeta‐potential and hydrophobicity, along with physicochemical, structural, and some techno‐functional attributes of sesame protein isolate at different pH levels (2.0–10.0) to explore new horizons for sesame protein in food and pharmaceutical applications.

## 2 Materials and Methods

### 2.1 Materials

Sesame seeds (10 kg) were procured from the farmland of Khorasan Province, Iran. The seeds with about 12% moisture content (wet basis) were dehydrated in an oven at 50°C, carefully cleaned, and ground into a powder using a pestle and mortar. Sunflower oil was obtained from national market. Unless otherwise stated, all reagents used in the work were of analytical grade and purchased from Sigma‐Aldrich Co. (St. Louis, MO).

### 2.2 Sesame Protein Preparation

Prior to protein extraction, the seed was defatted to avoid lipid oxidation according to our previous procedure with some modifications (Esmaeili et al. 2016; Rafe, Sadeghian, and Hoseini‐Yazdi 2017). Sesame seed was defatted twice using hexane solvent at a ratio of 1:4 at a setting of 400 rpm in a lab stirrer (Alfa, Model HS860, Iran) for 60 min and centrifuged (Universal 320R, Andreas Hettich GmbH & Co. KG, Germany) at 4000 *g* for 10 min at room temperature. The defatted sesame seed was air‐dried overnight under a hood, ground in a Moulinex miller (Model depose 00022, France), sieved through an 80 mesh screen (U.S. Standard sieve), packed in polyethylene bags, and stored at 5°C until the experiments. Defatted sesame contained approximately 7.5% moisture on wet basis.

Sesame protein was extracted according to Onsaard work with some modifications (Singharaj and Onsaard 2015). Defatted sesame seeds were dispersed in distilled deionized water at ratio 1:4, and the pH was adjusted to 9.5 using 2.0 N NaOH while stirring at ambient temperature for 60 min. In order to remove insoluble materials, the slurry was centrifuged at 3000 *g* for 15 min. Then, the supernatant was adjusted to pH 5.5 with 1 N HCl to precipitate proteins and centrifuged again at 3000 *g* for 15 min. The precipitate was washed with water at pH 5.5 and dispersed in a small amount of distilled water. The dispersed product was freeze‐dried (Zirbus VaCo 5, Laboratory Freeze Dryer, Germany) and stored at −5°C.

### 2.3 Physicochemical Properties

#### 2.3.1 Sesame Protein Yield

Sesame protein content was measured by the Kjeldahl method according to the AOAC procedure (920.87) with some modifications from the previous work (Esmaeili et al. 2016; Rafe, Sadeghian, and Hoseini‐Yazdi 2017), and the protein content was calculated by multiplying the nitrogen content by the protein conversion factor of 6.25. The other components including moisture (925.10), ash (923.03), fiber (920.86), and crude fat (920.39) were determined by the Association of Official Analytical Chemists methods (AOAC 2002), and the content of carbohydrates was calculated by subtracting the other amounts from 100.

Protein yield was also calculated by the following equation (Equation 1):

(1) $$\begin{array}{c} \text{Yield}\;\left(\%\right)\\ =\frac{\text{weight}\;\left(g\right)\;\text{of}\;\mathrm{SP}\times \text{Protein Content}\;\left(\%\right)\;\text{of}\;\mathrm{SP}}{10\;g\;\left(\text{weight of sesame seed}\right)\times \text{Protein Content}\;\left(\%\right)\;\text{of sesame seed}} \end{array}$$

#### 2.3.2 Bulk Density

Bulk density (*ρ*_b_), tapped/true density (*ρ*_t_), and compressibility index of the sesame protein were determined according to the European Pharmacopoeia. Twenty‐five grams of sesame protein powder was filled into a 100 mL graduated measuring cylinder without any compacting. The cylinder was tapped gently 400 times on a laboratory bench. Both *ρ*_b_ and *ρ*_t_ were expressed as g/mL. The compressibility index is the measure of the propensity of the powder to be compressed and determined according to Equation (2) as follows:

(2) $$\text{Compressibility index}=\frac{\left(V_{0}-V_{f}\right)}{V_{0}}\times 100$$

where *V*_0_ and *V*_f_ are the unsettled apparent volume and final tapped volume, respectively.

#### 2.3.3 Nitrogen Solubility, Dispersibility, and Wettability

Nitrogen solubility of the proteins at 1% (w/v) was determined by the method of Bradford over a pH range of 2.0–10.0 (Yüzer and Gençcelep 2023). The pH adjustment of the protein dispersions (1%, w/v) was performed using either 0.1 N HCl or NaOH. The suspensions were stirred over a magnetic stirrer for 1 h at room temperature (24°C). Then, the suspensions were centrifuged at 3000 *g* for 15 min. The protein analysis was performed on supernatant and bovine serum albumin (BSA) was used as a standard curve. Nitrogen solubility (NS) was expressed as % of nitrogen content of the sample according to Equation (3):

(3) $$\mathrm{NS}\;\left(\%\right)=\frac{\text{Nitrogen content in the supernatant}\;\left(\mathrm{mg}\right)}{\text{Total nitrogen in}\;a\;100\;\mathrm{mg}\;\text{sample}}\times 100$$

The Sharma procedure was used to measure the dispersibility of sesame protein at various pH levels (Sharma, Singh, and Sharma 2016). Three grams of sesame protein was dispersed in distilled water in a 50 mL measuring cylinder, and then the pH was adjusted using dilute HCl and NaOH solutions. After adding distilled water to reach a volume of 30 mL, the mixture was vigorously stirred and left to settle for 2 h. The dispersibility was then calculated using Equation (4):

(4) $$\text{Dispersibility}\left(\%\right)=\frac{\text{Total volume}-\text{Settled volume}}{\text{Total volume}}\times 100$$

The wettability was evaluated following the method outlined by the Sharma method (Sharma, Singh, and Sharma 2016). Two grams of powder was placed in a beaker with 80 mL of distilled water. The behavior of the powders on the water surface was observed immediately after adding the sample. After 30 min, the material was stirred on a magnetic stirrer to form a vortex reaching the bottom of the beaker. The stirring continued for 1 min, and the wettability was graded as excellent, good, fair, or poor based on the time and behavior of dispersion.

### 2.4 Surface Characteristics

#### 2.4.1 Zeta Potential and Particle Size

The particle size (nm) of the samples was determined using nanoparticle size analyzer‐zetasizer (Microtrac MRB, Nanotrac wave II). The device was employed by the dynamic light scattering. The zeta potential (mV) was measured with the same equipment based on the electrophoretic action of protein solutions. The protein sesame solutions (0.05%, w/v) were prepared by distilled water. Then, pH was adjusted from 2.0 to 10.0 with 0.1 N HCl or NaOH. Each sample was measured in triplicates (Yüzer and Gençcelep 2023).

#### 2.4.2 Surface Hydrophobicity

Surface hydrophobicity (*S*_0_) of the sesame protein was determined using the fluorescence 1‐anilino‐8‐naphthalenesulfonate (ANS) binding method (Chalamaiah et al. 2017). The 0.003% sesame protein solutions were prepared in milli Q water, and pH was adjusted to desired pH value (2–10) with 0.5 and 0.1 N HCl or NaOH. Fluorescence intensity (FI) was measured by adding 20 μL of ANS (8 mM) to 4 mL of the protein solution using a fluorescence spectrophotometer (Cary Eclipse, Model FP 6200; Jasco Inc., Maryland, USA) at excitation and emission wavelengths of 390 and 470 nm, respectively. *S*_0_ was also determined by centrifugation of protein solutions at 10,000 *g* for 15 min and adjusting pH to 7.0 at different protein concentrations from 0.0015% to 0.015%. The coefficient of linear regression analysis of the FI versus protein concentration (%) was used as an index of the protein surface hydrophobicity (*S*_0_).

### 2.5 Techno‐Functional Properties

#### 2.5.1 Water and Oil Holding Capacity

Water holding capacity (WHC) was determined by Asen and Aluko with slight modifications (Asen and Aluko 2022). 0.5 g sample was prepared by dispersing in 10 mL of 0.01 M phosphate buffer at pH 2.0–10.0. The dispersion was mixed for 1 min, allowed to stand for 30 min, and then centrifuged at 3000 *g* for 15 min at 25°C. The supernatant was decanted, and excess water was drained for 15 min; the gram of water retained per gram of sample was calculated.

The oil holding capacity (OHC) of protein samples was measured using the previously described method (Tang et al. 2003). Briefly, after mixing the protein powders with deionized water, the pH values were adjusted to 2.0–10.0 and stirred for 30 min. The solutions were freeze‐dried (Zirbus VaCo 5, Laboratory Freeze Dryer, Germany) to obtain protein samples. The freeze‐dried protein samples (1 g) were mixed with 10 mL sunflower oil for OHC by a stirrer for 30 min and centrifuged at 3000 *g* for 20 min. After centrifugation, the supernatant was carefully decanted, and the remaining pellets were weighed. The gram of oil retained per gram of sample was calculated.

#### 2.5.2 Emulsion Activity and Stability Index

The emulsifying properties of sesame protein were determined as a function of pH (2–10) (Esmaeili et al. 2016). Sesame protein dispersions (1%, w/v) were prepared with deionized water and adjusted to pH 2.0–10.0 using either 0.1 N HCl or NaOH. Soybean oil (20 mL) and 36 mL of 1% (w/v) sesame protein dispersion were mixed and homogenized at 12,700 rpm (M Tops SR30 homogenizer, South Korea) for 1 min. An aliquot (100 μL) of the emulsion was taken from the bottom of the container at 0 and 10 min after homogenization and mixed with 10 mL of 0.1% sodium dodecyl sulfate solution. The absorbance of the emulsions was measured at 500 nm with a spectrophotometer (AE Lab UV/VIS Spectrophotometer, AE‐S60‐4 U, China). The absorbance at 0 and 10 min was considered as the emulsion activity index (EAI) and the emulsion stability index (ESI) (Equation 5), respectively:

(5) $$\mathrm{ESI}=\frac{A_{0}\times t}{A_{10}-A_{0}}$$

where *A*_0_ and *A*_10_ are the absorbance at 0 min and after 10 min, respectively (Esmaeili et al. 2016).

#### 2.5.3 Foaming Capacity and Stability

Foaming capacity and foam stability were assessed by Esmaeili et al. (2016) with some modifications. The SPI solutions were prepared at (1%, w/v), and the pH was adjusted from 2.0 to 10.0 using either 0.1 NHCl or NaOH. The solutions were agitated in graduated plastic tubes at high speed 12,700 rpm with homogenizer for 1 min. Foam capacity (FC) was reported as (Equation 6):

(6) $$\;\mathrm{FC}\;\left(\%\right)=\frac{\text{Volume prior to agitation}-\text{Volume after agitation}}{\text{Volume prior to agitation}}\times 100$$

A similar procedure was used to determine the foam stability (FS), but the samples were allowed to stand for 20 min at room temperature and the residual foam volume was measured (Cano‐Medina et al. 2011). The following formula (Equation 7) was used to calculate FS:

(7) $$\mathrm{FS}\;\left(\%\right)=\frac{\text{Residual foam volume}\;}{\text{Totalfoam volume}\;}\times 100$$

### 2.6 Structure Behavior

#### 2.6.1 FTIR

FTIR analysis was conducted to determine the functional groups and interactions within the sesame protein samples. The samples were mixed with KBr, followed by pressing into pellets. The secondary structure of the protein was assessed using an FTIR spectrophotometer (Agilent Cary 630 FTIR, USA), with measurements taken across a wavelength range of 650 to 4000 cm^−1^ at resolution 4 cm^−1^ by using Agilent's Microlab software (Yüzer and Gençcelep 2023).

#### 2.6.2 Morphological Properties

The surface morphology of the sesame protein was investigated using a scanning electron microscopy (SEM, JSM‐700 LF JEOL, Japan) at a voltage acceleration of 20 kV. To enhance the electrical conductivity and obtain clearer images, a 10‐nm gold–palladium alloy coating (Quorum SC7620, UK) was applied to the sample surfaces before imaging. The scanning electron microscopy images were captured at magnification of 1000.

### 2.7 Statistical Analysis

Statistical analysis was performed on experiments conducted in triplicates, with data averaging and standard deviation calculation. Regression analysis and curves were depicted by using Sigmaplot 14.0, and the Duncan test was used to assess significant differences (*p* < 0.05) between the means for each treatment, utilizing SPSS version 26.

## 3 Results and Discussion

### 3.1 Protein Yield and Composition

The chemical composition of sesame meal and sesame protein isolate is provided in Table 1. The protein content of sesame meal and SPI were 41.96% and 90.60%, respectively. Furthermore, the protein extraction yield of SPI was 77.2%. These protein values were in line with previous work (Yüzer and Gençcelep 2023). The moisture, fat, and ash content of SPI were 4.93%, 0.46%, and 2.67%, respectively. The protein content of SPI (90.60%) was slightly lower than 94.60% (Achouri, Nail, and Boye, 2012), but higher than 90.50% (Sharma, Singh, and Sharma 2016; Saini, Sharma, and Sharma 2018), 86.33% (Fathi, Almasi, and Pirouzifard 2018), 84.4% (Zhao et al. 2012), 81.66% (Saatchi, Kiani, and Labbafi 2019), and 70.4% (Özdemir et al. 2022). These differences in sesame protein content may be attributed to various cultivars, seed composition, and extraction processes. The protein extraction yield was determined to be 77.20% based on the extraction conditions used in this study. The pH value of the protein was obtained 6.98, in agreement with previous findings (Yüzer and Gençcelep 2023). The carbohydrate and fiber content of sesame protein were obtained 1.34% and 0.68%, respectively. Similar results for the chemical composition of sesame protein have been reported, with moisture, fat, ash, fiber, and carbohydrate contents recorded at 6.86%, 0.08%, 2.08%, 0.03%, and 0.81%, respectively (Sharma, Singh, and Sharma 2016).

**TABLE 1:** Chemical composition and physical properties of sesame meal and sesame protein.

Sample	Chemical properties						Physical properties^a^			
	Moisture	Protein	Fat	Ash	Fiber	Carbohydrate	Wettability	*ρ*_b_	*ρ*_t_	CI
Sesame meal	7.57 ± 0.25	41.96 ± 0.12	1.70 ± 0.16	8.47 ± 0.12	14.76 ± 0.52	25.57 ± 0.15	Good	0.74 ± 0.01	0.83 ± 0.01	21.54 ± 0.89
Sesame protein	4.93 ± 0.21	90.60 ± 0.11	0.46 ± 0.01	2.67 ± 0.19	0.68 ± 0.12	1.34 ± 0.05	Good	0.66 ± 0.01	0.72 ± 0.01	19.22 ± 1.11

^a^ *ρ*_b_, *ρ*_t_, and CI are bulk density, tapped density, and compressibility index, respectively.

### 3.2 Bulk Density and Compressibility of Sesame Protein

Bulk density is a critical factor in powdery ingredients, affecting the flow‐ability and solubility of the product and significantly influencing packaging. The risk of oxidation also increases due to the presence of air in the intergranular spaces when the bulk density is high (Koc et al. 2011). Protein isolate is a key functional component of various high‐protein processed food products and thus affects the textural and nutritional properties of the foods; the digestibility of the nutrients must be known to fully evaluate the significance of the nutrient concentration. The values of the *ρ*_b_, *ρ*_t_, and compressibility index of the sesame protein isolate and sesame meal are provided in Table 1. Sesame meal showed higher bulk and tapped densities than that of the SPI. However, the *ρ*_b_ of SPI was lower than previous results (Sharma, Singh, and Sharma 2016), with a similar *ρ*_t_ (0.72 g/mL) being reported for SPI. The density values of sesame meal and SPI were higher than rice bran protein (0.55 g/mL) (Esmaeili et al. 2016), but lower than casein (0.89 g/mL), which makes it feasible for application in weaning foods (Chandi and Sogi 2007). The compressibility index, which measures a powder's ability to settle, was found to be 21.54 for sesame meal and 19.22 for SPI. When the *ρ*_b_ and *ρ*_t_ are closer in value, the interparticulate interactions are lower, resulting in greater flow‐ability. Thus, SPI has more flow‐ability than that of sesame meal due to the less value of CI. Similar behavior has been reported previously (Sharma, Singh, and Sharma 2016).

### 3.3 Nitrogen Solubility, Dispersibility, and Wettability

The functional properties of proteins are significantly influenced by their physicochemical and structural characteristics. Protein solubility, in particular, is a crucial factor for the functional properties of food proteins and serves as an important indicator of their potential applications. This is due to its significant impact on the texture, color, emulsification, foaming, and sensory attributes of food products (Haque, Timilsena, and Adhikari 2016). The solubility of proteins is influenced by factors such as temperature, conformation, ionic strength, and protein concentration, which in turn determine the charged amino acids responsible for protein solubility. Studies have shown a close relationship between protein solubility and its emulsifying and foaming properties (Wang et al. 1999).

**FIGURE 1:**
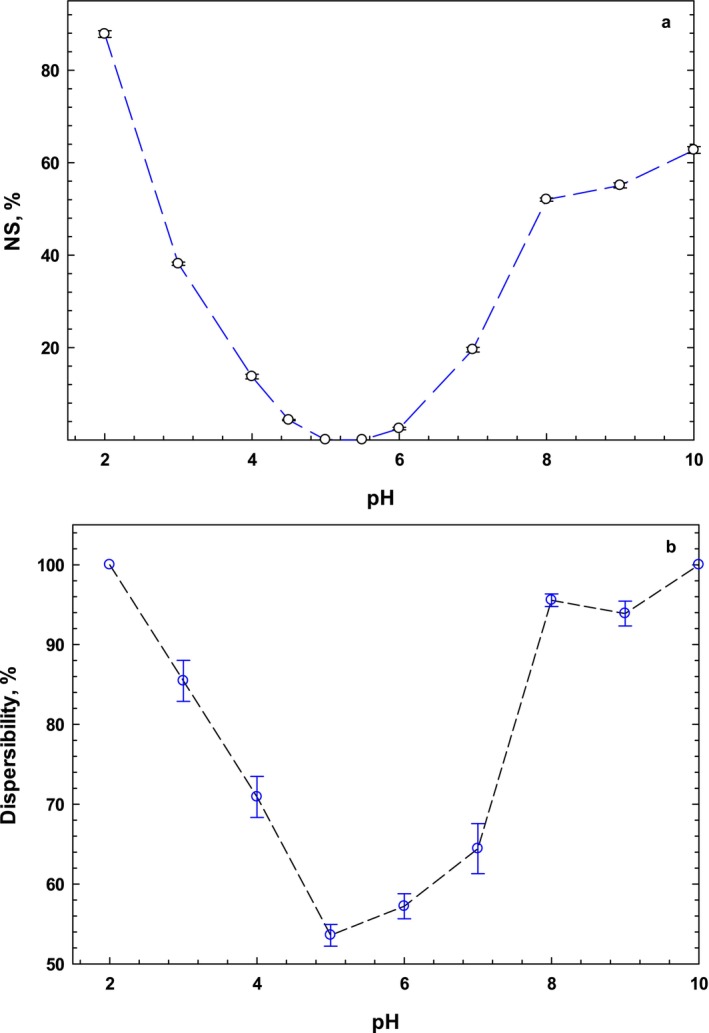
Protein solubility (a) and dispersibility (b) of the sesame protein isolate as a function of pH (2.0–10).

The sesame protein solubility profile at pH ranging from 2.0 to 10.0 is given in Figure 1a. The protein solubility in water showed a typical U‐shaped curve in which the lowest solubility was seen at isoelectric point (pI) at pH 5.0–5.5. This phenomenon is consistent with the principles of protein chemistry, where solubility is minimal at or near its pI due to reduced net charge and the resulting electrostatic interactions among protein molecules, leading to aggregation. The low nitrogen solubility near the pI of the protein can be attributed to a minimum net charge and its zwitterioninc state. In contrast, solubility increased beyond the pI, reaching 88% and 62% at acidic (pH 2.0) and alkaline (pH 10.0) conditions, respectively. Indeed, sesame protein underwent denaturation and hydrolysis under both acidic and basic conditions, resulting in increased solubility. Similar findings have been also reported by previous works (Zhao et al. 2012; Yüzer and Gençcelep 2023; Achouri, Nail, and Boye 2012; Khalid, Babiker, and Tinay 2003). It was also found the order of solubility of the sesame protein fractions was: albumin > globulin > prolamin, and glutelin (Idowu et al. 2021). Furthermore, nitrogen solubility of sesame protein was somewhat higher than that of rice brain proteins at pH values beyond the pI (Esmaeili et al. 2016). Moreover, the solubility of animal proteins, such as bovine serum albumin (BSA) (100%) and whey proteins (88.7%) (Voutsinas, Cheung, and Nakai 1983), is generally higher than that of plant proteins (with SPI solubility at neutral pH being ~45%). High solubility at extreme pH values (both acidic and alkaline) suggests that SPI can be effectively utilized in a variety of food products requiring solubilized proteins.

The hydration of food powders is considered along with their dispersibility and wettability properties. The dispersibility of SPI as a function of pH (2.0–10.0) is provided in Figure 1b. As seen, the dispersibility of SPI follows the same trend as solubility, with the lowest dispersibility observed at pH 5.0. The small particles of the SPI powder, due to the large surface area per mass, have shown lower dispersibility and wettability during the rehydration process. Consequently, lower wettability improved the rehydration rate of SPI, which is considered a desired technological property. In contrast, beyond the pI, higher dispersibility correlated with a reduced likelihood of agglomeration. Indeed, the presence of tiny particles negatively affected dispersibility due to reduced wettability during the rehydration process. Similar behavior has been reported for the spray‐dried and freeze‐dried powders of sesame protein (Özdemir et al. 2022). Although the lowest dispersibility of sesame protein isolate has been reported at pH 9.0, this study did not consider a wide pH range (Sharma, Singh, and Sharma 2016). The wettability of SPI powder is regarded as good since it is slightly wet upon contact with water, and after 30 min, the powder settled to the bottom, which is in agreement with the previous works (Sharma, Singh, and Sharma 2016).

### 3.4 Electrophoretic Mobility and Particle Size

**FIGURE 2:**
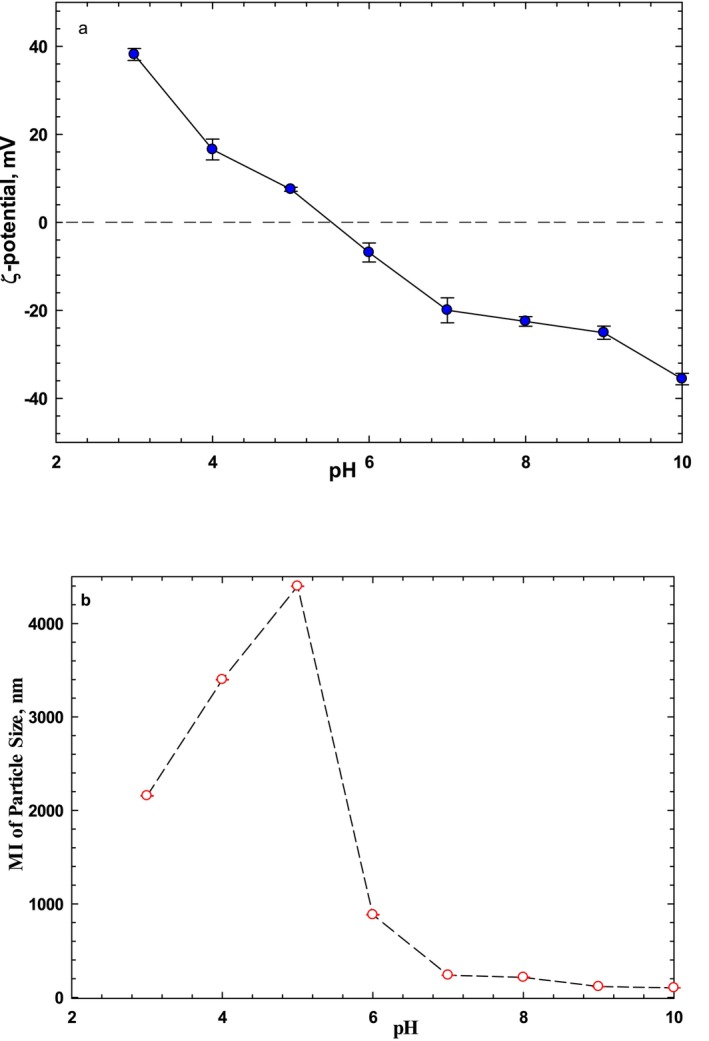
Effect of pH on zeta‐potential (a) and particle size (b) of sesame protein isolate.

The interactions of electric charges significantly affect the structure, stability, rheological behavior, texture, color, shelf life, and flavor of food systems. Zeta‐potential (ζ) is the most practical characteristic for evaluating charge density in biopolymers such as proteins, which principally affects emulsification, foaming, solubility, gelation, surface activity, stability, and interactions with other biopolymers (Cano‐Sarmiento et al. 2018). Surface charge density of sesame protein at pH range of 2.0–10.0 is provided in Figure 2a. The sesame protein had a neutral charge density at pH 5.0–6.0, which may be related to the isoelectric point around 5.5. As pH increased from 2.0 to 10.0, ζ‐potential gradually decreased from the positive to negative values, attributed to the gradual protonation of COO^−^ groups and deprotonation of NH^2+^ groups in the protein (Tang and Sun 2011; Rafe et al. 2022b). Plant protein isolates including soybean, chicken pea, lentil, as well as animal proteins such as whey protein, BLG, and casein have shown a similar pattern, where ζ‐potential was positive at pH 3.0, gradually declined to 0 adjusting pH to the pI (pH = 5.0–6.0), and then exhibited a negative trend with further increases in pH (Tang, Roos, and Miao 2023; Rafe et al. 2022b). Whereas the ζ‐potential of sesame protein was −19.97 ± 2.84 mV at pH 7.0, which aligns with the previous findings (−17.30 mV) (Yüzer and Gençcelep 2023). These findings indicate how the relationship between electrical charges in sesame protein isolate may affect the structure and stability of the product.

The hydrodynamic dimension as particle size of sesame protein has a particular effect on the functionality and emulsifying properties of proteins, depending on molecular size. The particle size of sesame protein as a function of pH is given in Figure 2b. As expected, the maximum molecular size occurred at around pH 5.0, corresponding to the pI and zero net charge, resulting in larger aggregates due to strong hydrophobic interactions. Similar hydrodynamic sizes for protein isolates, such as SPI (0.33 μm), soy protein isolate (0.32–0.68 μm), and bean protein isolate (100–200 nm), have been also reported in the literature (Mozafarpour et al. 2019).

### 3.5 Intrinsic Fluorescence and Surface Hydrophobicity

**FIGURE 3:**
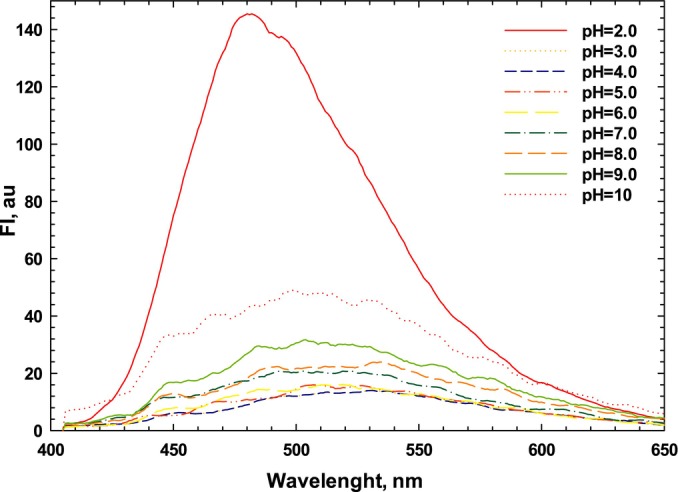
Fluorescence intensity of sesame protein isolates at varying pH levels.

Intrinsic fluorescence analysis can be used to determine the tertiary structure of proteins due to the predominant fluorescence in the emission region of tryptophan (Trp) found in proteins, permitting them to reflect the polarity changes of the environment (Zhang et al. 2022). Fluorescence intensity (FI) and maximum emission wavelength (*λ*_max_) of sesame protein isolate at varying pH levels are depicted in Figure 3. It is assumed that Trp is buried in a nonpolar environment if *λ*_max_ < 330 nm and is considered to be in a polar environment if *λ*_max_ > 330 (Chen et al. 2019). The results showed that the pH is effective on intrinsic fluorescence and the *λ*_max_ of protein showed a red shift when pH shifted away from 5.0 (close to IP), which shifted from pH = 5.0 to 9.0. Beyond the isoelectric point in both acidic and alkaline conditions, a red shift was also found, which can be attributed to the relocation of Trp residues from being hydrophobic to being more polar and hydrophobic, which generally has a negative impact on the FI of Trp groups (Ma et al. 2022). Furthermore, the maximum FI was found under acidic (pH 2.0) and alkaline (pH 10) conditions, with the lowest FI observed close to the pI at pH 5.0. Similar findings have been reported for plant protein isolates where maximum FI at alkaline conditions has been found compared with pH close to pI (Tang, Roos, and Miao 2023; Chen et al. 2019). This phenomenon can be related to aggregate formation at pH 5.0, which buries most intrinsic Trp residues, even though electrostatic repulsion effects can facilitate their migration to polar surfaces in an alkaline environment. These superior interface properties of SPI might be extracted from its greater exposure of hydrophobic groups.

Surface hydrophobicity (*S*_0_) is an indicator of hydrophobic amino groups on protein surfaces, which are closely related to diverse functional properties. A linear relationship between FI and protein concentration was observed, with a high regression coefficient (*r* = 0.99). Surface hydrophobicity was affected by pH, with *S*_0_ decreasing as pH increased. Therefore, the highest and lowest *S*_0_ were found at pH 2.0 and 10, respectively. The higher *S*_0_ value at low pH may be related to more exposure of hydrophobic residues resulting from the protein dissolution (Chang et al. 2015). The *S*_0_ of SPI was 38, which was higher than that of rice bran protein (33, 37) (Esmaeili et al. 2016) but lower than that of BSA (86.4) (Wang et al. 1999). It has a connection between surface hydrophobicity and the functional attributes of proteins, with higher *S*_0_ values corresponding to improved protein functionality. Studies have indicated that the surface hydrophobicity (*S*_0_) values of BSA, β‐lactoglobulin, whey, casein, and ovalbumin were 325, 426, 182, 28, and 6, respectively (Tang and Sun 2011). The *S*_0_ values of sesame protein were found to be greater than those of casein and ovalbumin, but lower than the other proteins. In general, surface hydrophobicity is a crucial factor influencing emulsifying properties, with emulsion activity primarily affected by *S*_0_ (Singharaj and Onsaard 2015; Tang and Sun 2011). Conversely, low surface hydrophobicity of sesame protein may hinder interactions between proteins and lipids, leading to a reduction in emulsifying properties (Wang et al. 1999). However, treatments involving heat and enzymatic processes can increase *S*_0_ and enhance the emulsion activity of sesame protein by exposing buried hydrophobic amino acids within the protein molecules.

### 3.6 Techno‐Functional Properties

#### 3.6.1 Water and Oil Holding Capacity

**FIGURE 4:**
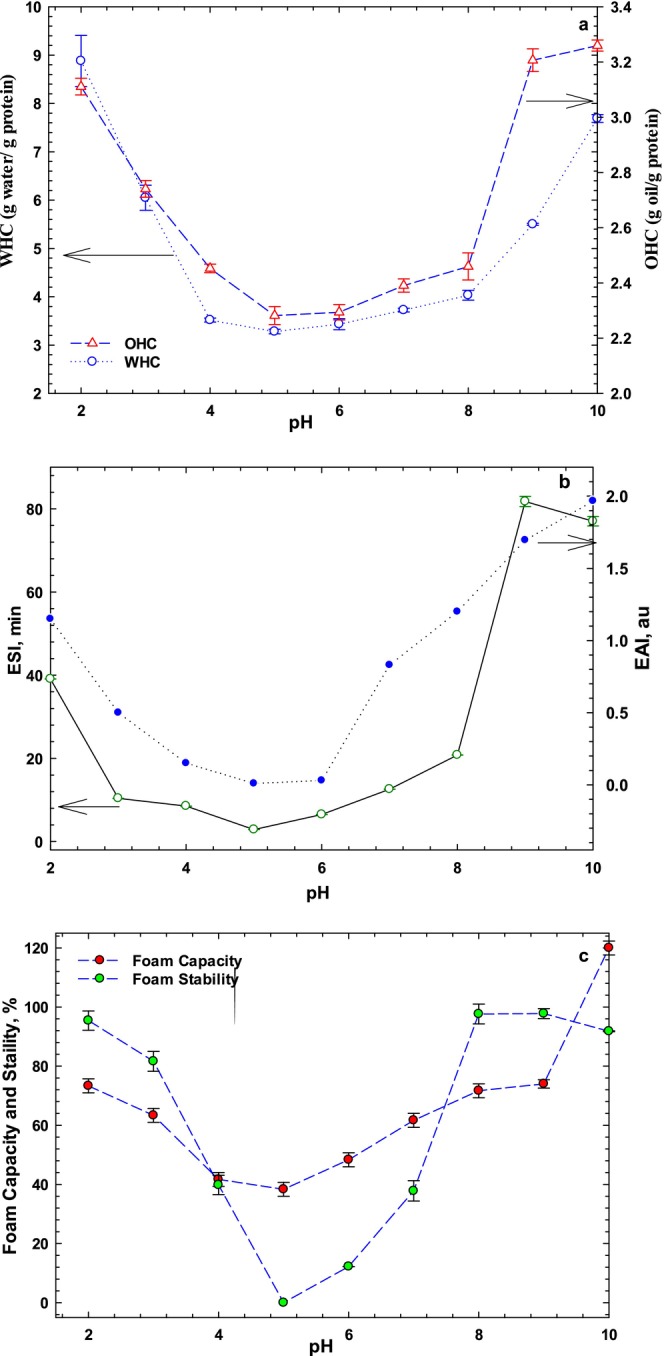
Effect of pH on functional properties of sesame protein isolate, WHC and OHC (a), ESI and EAI (b), and foam capacity and foam stability (c).

The water holding capacity (WHC) and oil holding capacity (OHC) as functions of pH are given in Figure 4a. WAC and OHC were 2.29–2.46 and 3.28–4.03 g/g, respectively, at neutral pH range (5.0–8.0). The lowest WHC and OHC were observed at pH around pI of sesame protein. Beyond the pI, a gradual increase in WHC and OHC was found, which may be resulted from a change in protein conformation that exposes more water binding's sites, protein polarity, ζ‐potential, solubility, and surface hydrophobicity, which are in line with the previous results. Furthermore, our findings on water and oil holding capacities exceeded those of the previous work (1.26 and 3.40 g/g) (Yüzer and Gençcelep 2023). Although SPI exhibited good WHC and OHC, the OHC was greater than WHC across the pH range, which can be related to the surface hydrophobicity of the sesame protein. Furthermore, OHC of SPI was less than soy protein isolate, and SPI had a higher OHC than chicken pea protein (2.81–4.04) (Saini, Sharma, and Sharma 2018). The potential of SPI is an exciting avenue, particularly its ability to enhance mouthfeel and flavor retention in various food products, including meat and dairy alternatives as well as bakery systems (Chen et al. 2019).

#### 3.6.2 Emulsifying Activity and Stability Indices

The emulsifying activity index is dependent on the peptides' diffusion at the interface and is a measure of the protein ability to provide adequate film to avoid fast association and assist in the dispersion of oil phase (Onsaard 2012). The emulsifying activity index (EAI) and emulsion stability index (ESI) of sesame protein as a function of pH (2.0–10.0) are shown in Figure 4b. The results showed that pH significantly affects the EAI and ESI of sesame protein, with the lowest EAI (0.01 ± 0.01) and ESI (2.87 ± 0.01 min) occurring at pH 5.0. Although EAI and ESI of sesame protein were increased at upper and lower pH level of pI of protein, the highest EAI and ESI were found at pH 10.0 and 2.0, respectively. Similar results have also been reported for emulsion activity of sesame protein isolates at pH 9 and 12 (Sharma, Singh, and Sharma 2016; Khalid, Babiker, and Tinay 2003), potentially due to increased Coulombic repulsions between neighboring droplets, combined with the high hydration of charged protein molecules that ultimately reduce interface energy and stabilize emulsion droplets. Indeed, protein solubility directly affected the emulsion activity of protein, which both reduce at the pI when the ζ‐potential tends to zero (see Sections 3.3 and 3.4). As a result, Coulombic forces induce the molecules to be more stable, and Brownian movement facilitates the suspension development than emulsion formation (Cano‐Medina et al. 2011). Furthermore, the emulsion activity and stability of sesame proteins have been higher in spray drying method than that of freeze‐drying technique, which emphasizes the effect of protein extraction methods on the emulsifying properties (Özdemir et al. 2022). Compared to BSA, which is considered a good emulsifier, it can be inferred that sesame protein has lower emulsifying properties (Wang et al. 1999). The EAI of SPI was found to be lower than that of BSA across all pH levels, possibly due to the lower *S*_0_ of sesame protein (38) compared to BSA (86.4), whey, casein, and ovalbumin (Esmaeili et al. 2016). Furthermore, sesame protein exhibited higher emulsifying properties than several other plant proteins such as rice bran protein (RBP) (37) (Rafe, Sadeghian, and Hoseini‐Yazdi 2017), which have emulsifying capacities ranging from 24% to 74% (Chandi and Sogi 2007). The alkaline extraction application not only effectively increased protein solubility but also enhanced the emulsifying properties of the extracted proteins. Heat processing during oil extraction can denature sesame protein, potentially leading to increased hydrophobicity and influencing its functional properties by exposing hydrophobic groups within the denatured proteins.

#### 3.6.3 Foaming Capacity and Stability

Foaming stability (FS) of protein is affected by film thickness, mechanical strength, and some other properties of the protein film. FS reveals foam's ability to retain its volume and shape over time, which is desirable in certain beverages and food products such as beer and baked goods. Foaming capacity (FC) and FS of SPI at various pH levels are depicted in Figure 4c. FC and FS after 10 min were 61.67% and 37.82%, respectively. High foam ability of protein (37.82%) may be associated with highly globular proteins resist to denaturation, linked to the high globular fraction of sesame protein (~67% globulin) (Singharaj and Onsaard 2015). It was also found the lowest FC and FS at pH 5.0, near the pI of SPI, while the greatest FS was seen at alkaline pH (8.0–9.0), which may be related to the *S*_0_ of SPI. However, the FC was gradually increased away from the pI, the most changes in FC occurred at alkaline conditions. Similar results have also been reported for plant proteins (Tang, Roos, and Miao 2023) and BLG (Rafe et al. 2022b, 2022a). While plant proteins have exhibited higher FS than dairy proteins due to the larger molecular weight of globulins, the latter form adsorption films with greater elasticity (Tang, Roos, and Miao 2023). Therefore, similar trends have been reported in FS and FC of RBP isolate (Esmaeili et al. 2016) and sesame protein (Khalid, Babiker, and Tinay 2003). Indeed, by increasing the net charge of proteins, the hydrophobic interactions are weakened and proteins become more flexible, leading to foam formation (Chen et al. 2019). High foamability of SPI can be attributed to good solubility, which enables it to dissolve in aqueous phase and quickly develop a cohesive layer at the interface layer, which induces the low surface tension. In contrast, high intermolecular cohesiveness and elasticity enables the SPI to produce stable foams (Tang et al. 2003; Rafe et al. 2022b).

### 3.7 Structural Properties

**FIGURE 5:**
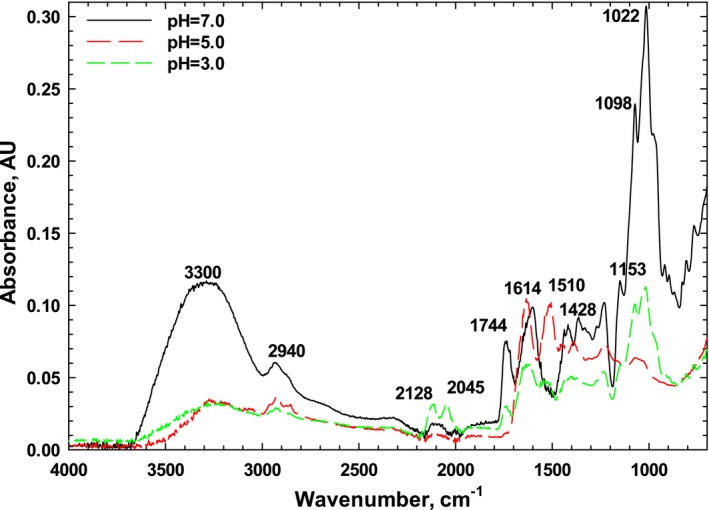
FTIR spectra of sesame protein isolate at various pH levels (3.0, 5.0, and 7.0).

The chemical structure of the SPI at pH values 3.0, 5.0, and 7.0, representative of acidic, pI, and neutral pH, was determined by using Fourier transform infrared spectroscopy (FTIR), and the peaks are illustrated in Figure 5. FTIR of sesame protein revealed functional groups of protein spectrum, i.e., Amide I (1744 cm^−1^) and Amide II (1614 cm^−1^), which is attributed to –NH stretching vibrations, typical of the backbone of protein representing β sheets (Saini, Sharma, and Sharma 2018). The peak at around 1428 cm^−1^ corresponds to CN stretching and NH bending. The characteristic peaks in Amid I and Amid II were predominantly due to N‐H stretching vibrations. Important bands related to various peptide conformations were also found below 1400 cm^−1^, representing α‐helix and β sheets (Amide III). In particular, the density of the Amid I group was stronger than that of Amid II and Amid III. It has been also observed the Amid I, Amid II, and Amid III bands of sesame protein concentrate at 1635, 1518, and 1234 cm^−1^, respectively (Saatchi, Kiani, and Labbafi 2019). Peaks observed at 767 cm^−1^ represent Amide IV which attributes to OCN bending, while Amide V was represented by peaks at 800 cm^−1^, indicating out‐of‐plane NH bending. These results closely align with FTIR results obtained for sesame protein isolate in the current study, corroborating findings from other investigations (Saini, Sharma, and Sharma 2018).

Due to the presence of charged groups in SPI, its structure was affected by the pH and ionic strength of the environment. Under acidic conditions (pH 3.0 and 5.0), the peaks in the range of 3300–3400 cm^−1^ shifted to lower frequencies, possibly due to unfolding, hydrophobic chain reactions, molecular interactions between positively and negatively charged groups, and formation of hydrogen bands. Furthermore, with pH reduction, α‐helix and β‐sheet structures decreased while more random coils developed. At the isoelectric point of SPI, the intermolecular β‐sheet at 1640 cm^−1^ was relatively prominent, and the band intensity at 1647 cm^−1^ (pH = 3.0) decreased compared to pH 7.0. Hence, an increase in intermolecular β‐sheet structures at pH 5.0 and more α‐helices at pH 7.0 was observed. Similar findings regarding the pH effect on the protein structure have been reported for BLG (Fang and Dalgleish 1997; Rafe and Razavi 2015) and *Spirulina platensis* peptides (Akbarbaglu et al. 2022).

### 3.8 Microstructural Properties

**FIGURE 6:**
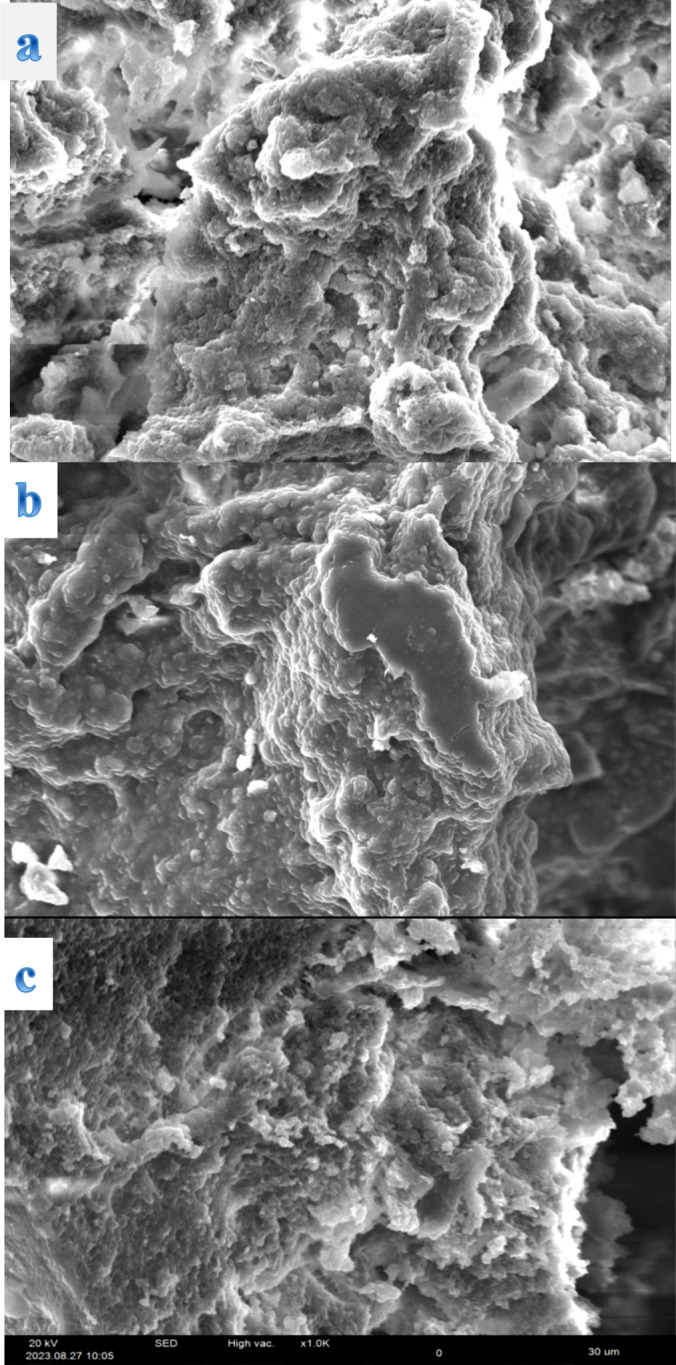
SEM images of sesame protein isolates at selected pH 3.0 (a), 5.0 (b), and 7.0 (c) (magnification 1000×, 20 kV).

The structural morphology of sesame protein isolate was examined with the aid of scanning electron microscopy at three selected pH levels (3.0, 5.0, and 7.0), representative acidic, isoelectric point, and neutral pH, at magnifications 1000 (Figure 6). The particles of the sesame protein isolate powder exhibited irregular shapes and lack of uniformity. The surface appeared rough, and wrinkled, with particles being more amorphous and agglomerated. It has been reported that the alkali extraction and isoelectric precipitation techniques employed in sesame protein isolates transform the protein's microstructure into a compact structure with a wrinkled surface (Idowu et al. 2021; Koc et al. 2011). Furthermore, the molecular structure of the SPI became more compact under acidic conditions due to protein denaturation (Figure 6b). SEM images showed aggregated and spherical particles, akin to the crumbled, multi‐layered sheet‐like structures reported for lentil protein isolate (Joshi et al. 2011).

At pH 3.0, the most pronounced changes in morphology were observed, with particles exhibiting a more pronounced aggregation, indicating the influence of acidic conditions on protein interactions. These aggregates suggest that the proteins are clumping together due to increased ionic interactions and reduced solubility in the presence of acid. In contrast, at pH 5.0, aligning with the isoelectric point, a notable reduction in particle size and an increase in spherical shapes were identified. This transition emphasizes the significance of the pH environment in modulating the structural characteristics of the sesame protein isolate, as the electrostatic repulsion between charged particles diminishes, allowing closer aggregation. At neutral pH, the structures appeared more diverse, indicating a mixture of both compact and amorphous forms. The surface characteristics remained rough, but there was a tendency towards more distinct particle separation compared to the acidic environment. This variation points to the delicate balance between protein solubility and aggregation, integral to defining the functional properties of sesame protein isolate. Previous studies have shown that the protein's texture and functionality, such as emulsification and foaming capacities, are closely linked to its morphological characteristics (Yan et al. 2024). The variations in surface morphology and particle arrangement at different pH levels underline the adaptability of sesame protein isolate in various food applications, where such structural features can significantly influence its functional properties. Additionally, the relationships observed among pH, morphology, and protein denaturation draw attention to the potential for optimizing processing parameters to enhance the utilization of sesame protein isolates in food formulations and nutritional applications.

## 4 Conclusion

The protein extraction yield of sesame seed was 77.2%, making it suitable as a natural plant protein for food and pharmaceutical applications. SPI has a pI of ~5.5, where protein solubility is low due to its minimum net charge and zwitterionic state. However, SPI showed the lowest dispersibility, water and oil holding capacity, and emulsifying and foaming properties at pH values 5.0–5.5. It demonstrated high flowability due to a lower compressibility index, allowing for larger aggregate formation through strong hydrophobic interactions, ultimately improving the rehydration rate of SPI, which is a desired technological property. In contrast, beyond pI, SPI underwent denaturation and hydrolysis at both acidic and alkaline pH levels, resulting in increased solubility and other techno‐functional properties, including FI, WHC, OHC, EAI, FC, and FS. Furthermore, at neutral pH, SPI exhibited ζ‐potential of −19.97 mV, revealing solubility of ~45%, hydrodynamic size of 0.33 μm, and surface hydrophobicity of 38, which provide suitable techno‐functional properties for food applications. In addition, SPI exhibited high WHC and OHC, with OHC exceeding WHC in the pH range, improving mouthfeel and flavor retention in various food products, including meat and bakery items. FTIR and SEM findings confirmed the techno‐functionality of sesame protein. FTIR data exhibited an increase in intermolecular β‐sheet structures at pH 5.0 and more α‐helices at pH 7.0. variations in surface morphology and particle arrangement at different pH levels underline the adaptability of SPI in various food applications. Given these properties, the interaction of SPI with other food components such as hydrocolloids to entrap bioactive ingredients may present an interesting avenue for future work. Notably, SPI exhibited greater foam stability than dairy proteins due to the higher molecular weight of globulins, which form elastic adsorption films, positioning it as a candidate for enhancing foaming in food aqueous systems. Further research into the structure–property relationships of sesame protein at different pH levels, along with its interplay with other food components, will be crucial in unlocking its full potential for diverse industrial applications.

## Author Contributions

**Azade Ghorbani:** conceptualization (equal), data curation (equal), formal analysis (equal), investigation (equal), methodology (equal). **Ali Rafe:** project administration (lead), resources (lead), supervision (equal), validation (lead), writing – review and editing (equal). **Mohammad Ali Hesarinejad:** project administration (equal), software (equal), visualization (equal). **Jose M. Lorenzo:** formal analysis (equal), validation (equal), writing – review and editing (equal).

## Disclosure

The authors have nothing to report.

## Conflicts of Interest

The authors declare no conflicts of interest.

## Data Availability

The data that support the findings of this study are available on request from the corresponding author.
